# 4-Hydroxyderricin inhibits osteoclast formation and accelerates osteoblast differentiation

**DOI:** 10.1007/s10616-018-0236-2

**Published:** 2018-11-24

**Authors:** Hiromi Hagiwara, Kyoko Nakata, Hitoshi Miyazaki, Sanae Maehashi, Yuki Komiyama, Rieko Aida, Shigeki Yoshida, Daichi Kokubu, Keitaro Hagiwara, Kaoru Yoshida

**Affiliations:** 1 Faculty of Biomedical Engineering, Toin University of Yokohama, 1614 Kurogane-cho, Aoba-ku, Yokohama, 225-8503 Japan; 2 Faculty of Life and Environmental Sciences, University of Tsukuba, 1-1-1 Tennodai, Tsukuba, 305-8572 Japan; 3 Healthcare Systems Co., Ltd, 2-22-8 Chikusa-ku, Nagoya, 464-0858 Japan

**Keywords:** 4-Hydroxyderricin, Polyphenol, Osteoblast, Osteoclast, Bone

## Abstract

4-Hydroxyderricin (4-HD) is a major polyphenol of *Angelica keiskei* (Japanese name Ashitaba), exhibiting anti-allergic, anti-diabetic, anti-oxidant, and antitumor effects. The present study was designed to evaluate the effects of 4-HD on bone formation and maintenance by using cultured osteoclasts and osteoblasts. 4-HD did not affect cell proliferation of stromal ST2 cells and preosteoblast MC3T3-E1 cells at concentrations of 1–10 μM. This compound inhibited the formation of multinucleated osteoclasts from mouse splenic cells, and we identified a molecular pathway of osteoclast differentiation mediated by 4-HD, which led to inhibition of the expression of receptor activator of nuclear factor-κB ligand and macrophage-colony stimulating factor in ST2 cells. By contrast, 4-HD enhanced indices of osteoblast differentiation, such as alkaline phosphatase activity and calcium deposition by osteoblastic MC3T3-E1 cells, at concentrations of 1–10 μM. Furthermore, we found that 4-HD at 1 μM attenuated H_2_O_2_ levels in MC3T3-E1 cells. Our findings indicate that 4-HD may have critical effects on bone formation and maintenance.

## Introduction

Both the formation and maintenance of bone are controlled by bone-resorbing osteoclasts and bone-forming osteoblasts. Osteoclasts are multinucleated giant cells with the ability to resorb mineralized tissues. They are formed from hematopoietic cells of the monocyte/macrophage lineage (Udagawa et al. 1990). The development of osteoclasts in culture is strictly dependent on support provided by osteoblasts and/or stromal cells (Udagawa et al. 1990). The formation and activation of osteoclasts are controlled by the combined actions of receptor activator of nuclear factor-κB ligand (RANKL) and macrophage colony-stimulating factor (M-CSF). Here, we formed multinucleated osteoclasts from splenic cells by co-culture with stromal ST2 cells that had been stimulated by activated vitamin D_3_ (Hagiwara et al. 2008, 2015; Notoya et al. 2007). Bone formation involves a complex series of events that include the proliferation and differentiation of osteoprogenitor cells, resulting in the formation of a mineralized extracellular matrix. The deposition of calcium and the sequential expression of type I collagen, alkaline phosphatase, and osteocalcin are known as markers of osteoblastic differentiation. Several model systems have been developed for studying the proliferation and differentiation of bone-forming cells in vitro and the molecular biology of the mineralization process, such as preosteoblastic cells from mouse calvariae (MC3T3-E1 cells) and osteoblast-like cells from rat calvariae (Bredford et al. 1993; Hagiwara et al. 1996; Liu et al. 1994; Stein et al. 1990). An imbalance of activities between osteoclasts and osteoblasts leads to bone metabolic diseases such as osteoporosis and osteopetrosis.

**Fig. 1:**
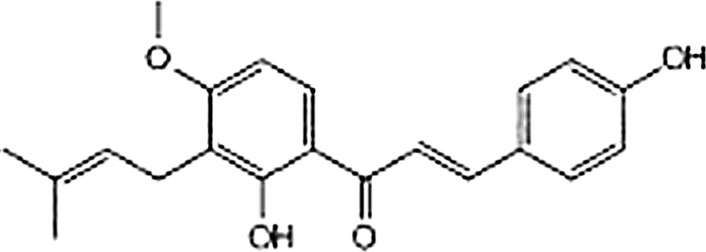
Structure of 4-HD

4-Hydroxyderricin (4-HD) (Fig. 1) is more abundant as aglycone forms, with relative abundances of 1.5% in the stem exudates of *Angelica keiskei* (Japanese name, Ashitaka). A number of studies have shown that this compound possess biological properties, including antidiabetic (Li et al. 2016; Zhang et al. 2015; Enoki et al. 2007), anti-inflammatory (Yasuda et al. 2014; Yadav et al. 2011) and antitumor (Sumiyoshi et al. 2015; Akihisa et al. 2011; Kimura et al. 2004; Okuyama et al. 1991) activities. However, little information is available on the effects of 4-HD on bone metabolism. The present study was designed to evaluate the in vitro effects of 4-HD on the formation and maintenance of bone by using cultured mouse cells. Our results indicate that 4-HD is useful in the prevention and treatment of osteoporosis.

## Materials and methods

### Purification of 4-HD from *Angelica keiskei*

Chalcone-rich powder of *Angelica keiskei* (Ashitaba in Japanese) was purchased from Japan Bio Science Laboratory Co., Ltd (Osaka, Japan). The powder (2 g) was extracted with 100 ml of ethyl acetate at room temperature for 1 h. The extract was subjected to silica gel chromatography equilibrated with hexane/ethyl acetate (3:1, v/v), and eluted with hexane/ethyl acetate (1:1, v/v). The eluate containing 4-HD was subjected to ODS chromatography (Cosmosil C-18 OPN, Nacalai Tesque, Kyoto, Japan) equilibrated with 100% methanol to give pure 4-HD.

### Formation of osteoclastic cells

Multinucleated osteoclastic cells were formed from spleen cells by co-culture with ST2 cells (RIKEN Cell Bank, Tsukuba, Japan) that had been stimulated by 100 nM 1α, 25-dihydroxy-vitamin D_3_ [1α, 25(OH)_2_ vitamin D_3_] (Wako Pure Chemical Industries, Ltd., Osaka, Japan). ST2 cells (2 × 10^4^ cells/well) in 48-well plates (area of each well, 0.75 cm^2^) were pre-cultured with 100 nM 1α, 25(OH)_2_ vitamin D_3_ for 1 day to express RANKL. Spleen cells were collected from the splenic tissues of 6-weeks-old male ddY mice (Sankyo Labo Service, Tokyo, Japan). Erythrocytes contaminating the spleen cell fraction were eliminated by adding 0.83% ammonium chloride in 10 mM Tris–HCl (pH 7.4) to the cell pellet. Mouse spleen cells (1 × 10^5^ cells/well) were spread on ST2 cell layers in 48-well plates and cultured in α-Modified minimum essential medium (α-MEM: Technologies, Inc. Grand Island, NY, USA), supplemented with 10% fetal bovine serum (Moregate BioTech, Bulimba, Australia), 50 units mL^−1^ penicillin and 50 μg mL^−1^ streptomycin (Life Technologies, Inc., Grand Island, NY, USA), in a humidified atmosphere of 5% CO_2_ in air at 37 °C for 7 days. Cultures were maintained at 37 °C in a humidified atmosphere of 5% CO_2_ in air. Fresh medium, 100 nM 1α, 25(OH)_2_ vitamin D_3_ and 4-HD were supplied at 2-days intervals. The Institutional Animal Care and Use Committee of Toin University of Yokohama approved all animal protocols and procedures.

Multinucleated osteoclastic cells formed were fixed in 3.7% formaldehyde for 5 min and then in a mixture of ethanol and acetone (1:1; v:v) for 1 min. These cells were then stained for tartrate resistant acid phosphatase (TRAP) activity (Udagawa et al. 1990). TRAP activity is a marker of multinucleated osteoclasts. TRAP-positive multinucleated cells (five or more nuclei) were counted under a microscope (IX70; Olympus, Tokyo, Japan).

### Osteoblastic cell cultures

Preosteoblastic MC3T3-E1 cells were obtained from RIKEN Cell Bank (Tsukuba, Japan). Cells were maintained in a 55-cm^2^ dish in α-Modified minimum essential medium (α-MEM), supplemented with 10% fetal bovine serum, 50 units mL^−1^ penicillin and 50 μg mL^−1^ streptomycin, in a humidified atmosphere of 5% CO_2_ in air at 37 °C. After reaching 70% confluence, cells were detached by treatment with 0.05% trypsin, replated in either 55-cm^2^ dishes or 12-well plates (area of each well, 3.8 cm^2^) at a density of 1 × 10^4^ cells/cm^2^, and grown in α-MEM supplemented with 10% fetal bovine serum, 50 units mL^−1^ penicillin, 50 μg mL^−1^ streptomycin, 5 mM β-glycerophosphate, and 50 μg mL^−1^ ascorbic acid. Fresh medium and 4-HD were supplied to cells at 2-days intervals. MC3T3-E1 cells formed nodules, and mineralization of nodules was observed after cultivation for 2–3 weeks.

### Toxicity of 4-HD for cells

ST2 cells and MC3T3-E1 cells were replated in 96-well plates (area of each well, 0.32 cm^2^) at a density of 2.5 × 10^3^ cells/cm^2^ and grown in α-MEM supplemented with 10% fetal bovine serum, 50 units mL^−1^ penicillin, 50 μg mL^−1^ streptomycin, and 4-HD at various concentrations. After subculture for 53 or 74 h, the cell layers were washed with RPMI 1640 medium (Life Technologies, Inc., Grand Island, NY, USA). 3-[4, 5-Dimethylthiazol-2-yl]-2, 5-diphenyltetrazolium bromide (MTT; DOJINDO, Kumamoto, Japan) reagent (0.5 mg mL^−1^ RPMI 1640) was added to each well, followed by incubation for 4 h for formazan formation. After the medium was removed, dimethyl sulfoxide was added to each well to dissolve the formazan, and absorbance was measured at 570 nm.

### Measurement of intracellular reactive oxygen species

Intracellular reactive oxygen species were measured using the oxidant-sensitive probe 2′, 7′-dichlorofluorescin diacetate (DCFH-DA). MC3T3-E1 cells (4 × 10^4^ cells/dish) in 3.5-cm dishes were cultured for 48 h with subsequent 48-h incubation with 4-HD. Thereafter, cells were incubated with 50 μM DCF-DA for 30 min followed by a 30-min incubation with 500 μM H_2_O_2_ and washed with Hank’s Balanced Salt Solution. Fluorescence emission was detected by confocal laser scanning microscopy at excitation and emission wavelengths of 488 and 505 nm, respectively. Images were analyzed using a confocal scanning system (TCP SP2; Leica, Tokyo, Japan).

### Measurement of alkaline phosphatase activity

MC3T3-E1 cells were subcultured in 12-well plates (3.8 cm^2^/well) in α-MEM containing 10% fetal bovine serum, 5 mM β-glycerophosphate, and 50 μg mL^−1^ ascorbic acid. After the cells had reached confluence (day 3), 4-HD was added to cultures at various concentrations for 9 days. Cells were washed with 10 mM Tris–HCl, pH 7.2, and were sonicated in 1 mL of 50 mM Tris–HCl (pH 7.2) containing 0.1% Triton X-100 and 2 mM MgCl_2_ for 15 s with a sonicator (Ultrasonic Disruptor UD-201; Tomy Co., Tokyo, Japan). Alkaline phosphatase activity was determined using an established technique with *p*-nitrophenyl phosphate as the substrate. Protein concentrations were determined using BCA protein assay reagent (Pierce Chemical Co., Rockford, IL, USA) with bovine serum albumin as a standard.

### Quantitation of calcium deposition

MC3T3-E1 cells were subcultured in α-MEM containing 10% fetal bovine serum, 5 mM β-glycerophosphate, and 50 μg mL^−1^ ascorbic acid. After the cells had reached confluence (day 3), 4-HD was added at various concentrations to the culture medium and cells were subcultured for 11 days. The amount of calcium, deposited as hydroxyapatite in the cell layer, was measured as follows: Layers of cells in 12-well plates (3.8 cm^2^/well) were washed with PBS and incubated overnight with 1 mL of 2 N HCl with gentle shaking. Ca^2+^ ions in the samples were quantitated by the *o*-cresolphthalein complexone method with a Calcium C kit (Wako Pure Chemical Industries). This kit is specific for Ca^2+^ ions and has a detection limit of 1 μg mL^−1^. The solution of Ca^2+^ ions (20 mg dL^−1^) provided in the kit was used as the standard solution.

### Real-time polymerase-chain-reaction (PCR)

The mRNA expression of RANKL and M-CSF in ST2 cells treated with 100 nM 1α, 25(OH)_2_ vitamin D_3_ was examined by real-time PCR. The RNeasy Mini Kit (Qiagen K.K., Tokyo, Japan) was used to extract RNA from cells that had been exposed to 4-HD for 2 days. Total RNA (1 μg) was reverse transcribed using the Transcriptor First Strand cDNA Synthesis Kit (Roche, Tokyo, Japan) with random primers in a 20-μL reaction mixture according to the manufacturer’s protocol. Quantitative polymerase chain reaction analysis was performed with LightCycler 480 System II (Roche) and LightCycler 480 SYBR Green I Master (Roche). PCR (95 °C for 10 s, 55 °C for 10 s, and 72 °C for 10 s, for 45 cycles) was performed using specific primers (sense primer, 5′-TGTACTTTCGAGCGCAGATG-3′, and antisense primer, 5′-CCCACAATGTGTTGCAGTTC-3′) for mouse RANKL, (sense primer, 5′-TTGCCAAGGAGGTGTCAGAA-3′, and antisense primer, 5′-TATTGGAGAGTTCCTGGAGC-3′) for mouse M-CSF, and (sense primer, 5′-ACTTTGTCAAGCTCATTT-3′, and antisense primer, 5′-TGCAGCGAACTTTATTG-3′) for mouse glyceraldehyde-3-phosphate dehydrogenase (GAPDH). GAPDH was used as an internal standard for normalization of each sample.

### Statistical analysis

Numerical data have been expressed as mean ± S.D. values of the results from three to four cultures, and the significance of differences was analyzed by using ANOVA (Dunnett’s test) or Tukey–kramer. Statistical significance was set at *P* < 0.05. Experiments were repeated independently in triplicate and the results were qualitatively identical in every case. Results from representative experiments are shown.

## Results

### Toxicity of 4-HD

**Fig. 2:**
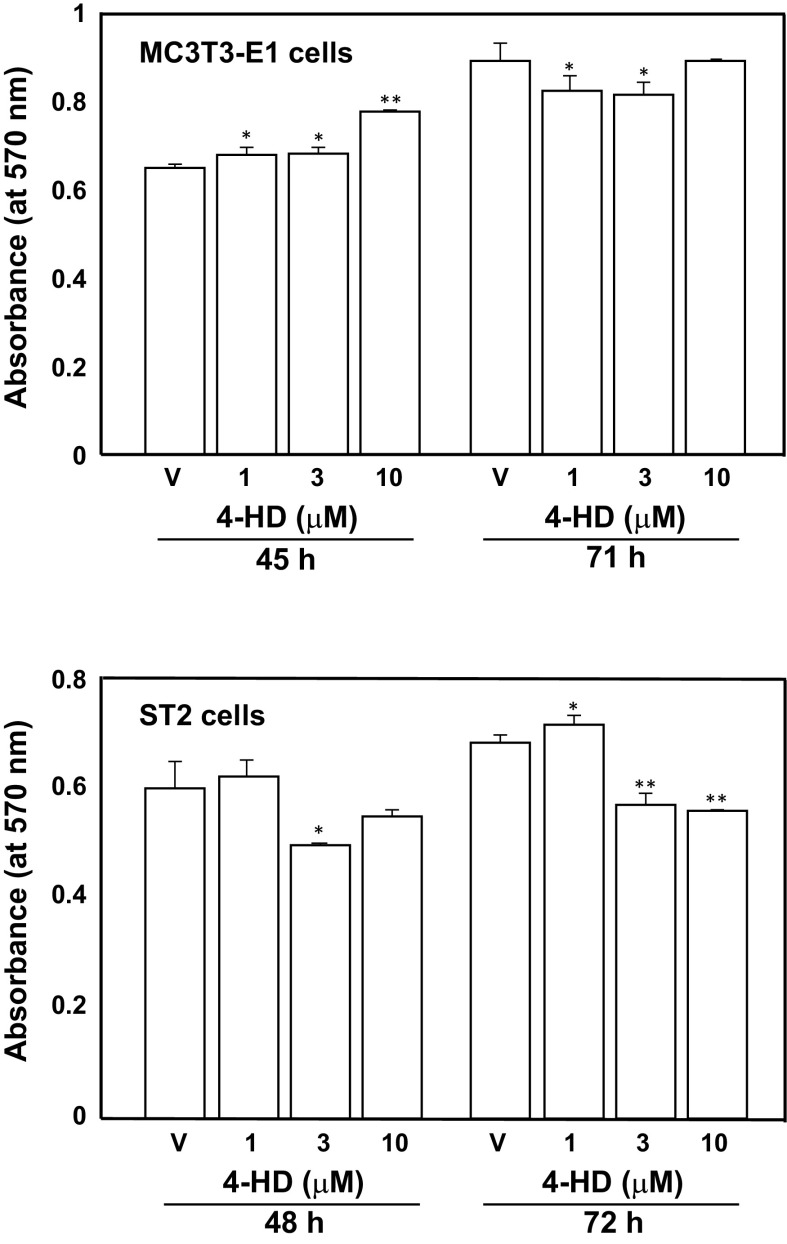
Effects of 4-HD on osteoblastic cell viability. MC3T3-E1 cells and ST2 cells (each 2.5 × 10^3^ cells/well; 96-well plates) were exposed to 4-HD at various concentrations (1–10 μM) and were subcultured for indicated periods. After treatment with 4-HD, the cells were treated with MTT (50 μg/well) for 4 h, and the absorbance at 570 nm was measured. The values represent the mean ± S.D. of results from three wells. Data are representative of results from three separate experiments. *P < 0.05 versus vehicle (V) and **P < 0.01 versus V

We evaluated the toxicity of 4-HD for MC3T3-E1 cells and ST2 cells by using the MTT assay. As shown in Fig. 2, the viability of MC3T3-E1 cells and ST2 cells was significantly increased and decreased at some concentrations. However, its disparity is not great. Furthermore, exposure of MC3T3-E1 cells and ST2 cells to 4-HD at 10 μM did not affect cell morphological features (data not shown).

### Effects of 4-HD on the formation of multinucleated osteoclasts

**Fig. 3:**
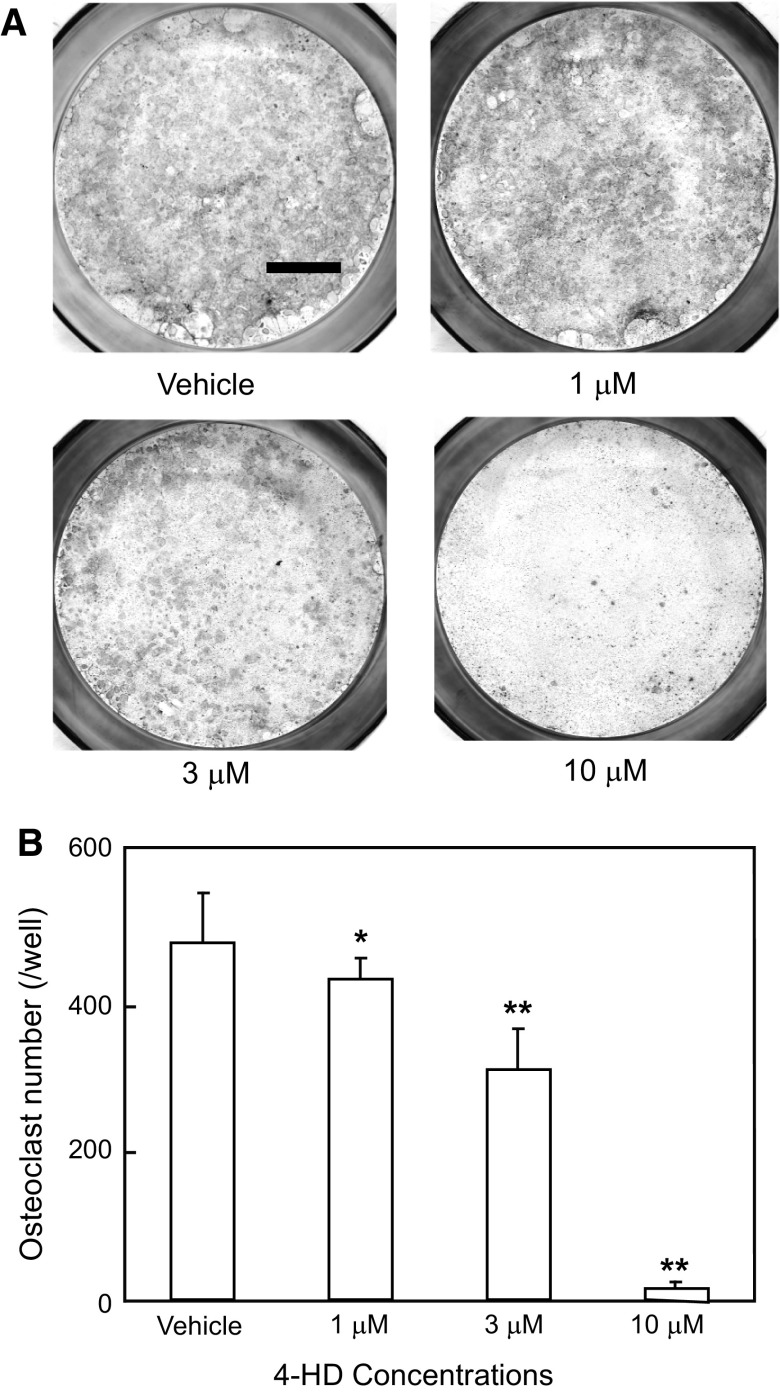
Effects of 4-HD on osteoclast formation. Osteoclasts were formed from mouse spleen cells. **a** Typical results of staining for the detection of TRAP activity. 4-HD was added to cultures at the indicated concentrations. Cultured cells were then stained for TRAP activity on day 7. Bar = 5 mm. **b** 4-HD was added to cultures at the indicated concentrations. Cultured cells were then stained for TRAP activity on day 7. TRAP-positive multinucleated cells (five or more nuclei) were counted under a microscope. Columns and bars show mean ± S.D. values of the results from five wells. Data are representative of the results of three separate experiments. *P < 0.05 versus vehicle (V), **P < 0.01 versus vehicle (V)

Multinucleated osteoclastic cells were formed from mouse splenic cells in co-culture with ST2 cells which had been stimulated by 1α, 25(OH)_2_ vitamin D_3_. Figure 3a shows representative results for the detection of TRAP activity in multinucleated osteoclastic cells treated with 4-HD at the indicated concentrations. Formation of TRAP-positive multinucleated osteoclastic cells was dose-dependently inhibited by the addition of 4-HD (Fig. 3b). Exposure of 10 μM 4-HD completely inhibited the formation of multinucleated osteoclastic cells relative to control cultures treated with the vehicle alone.

**Fig. 4:**
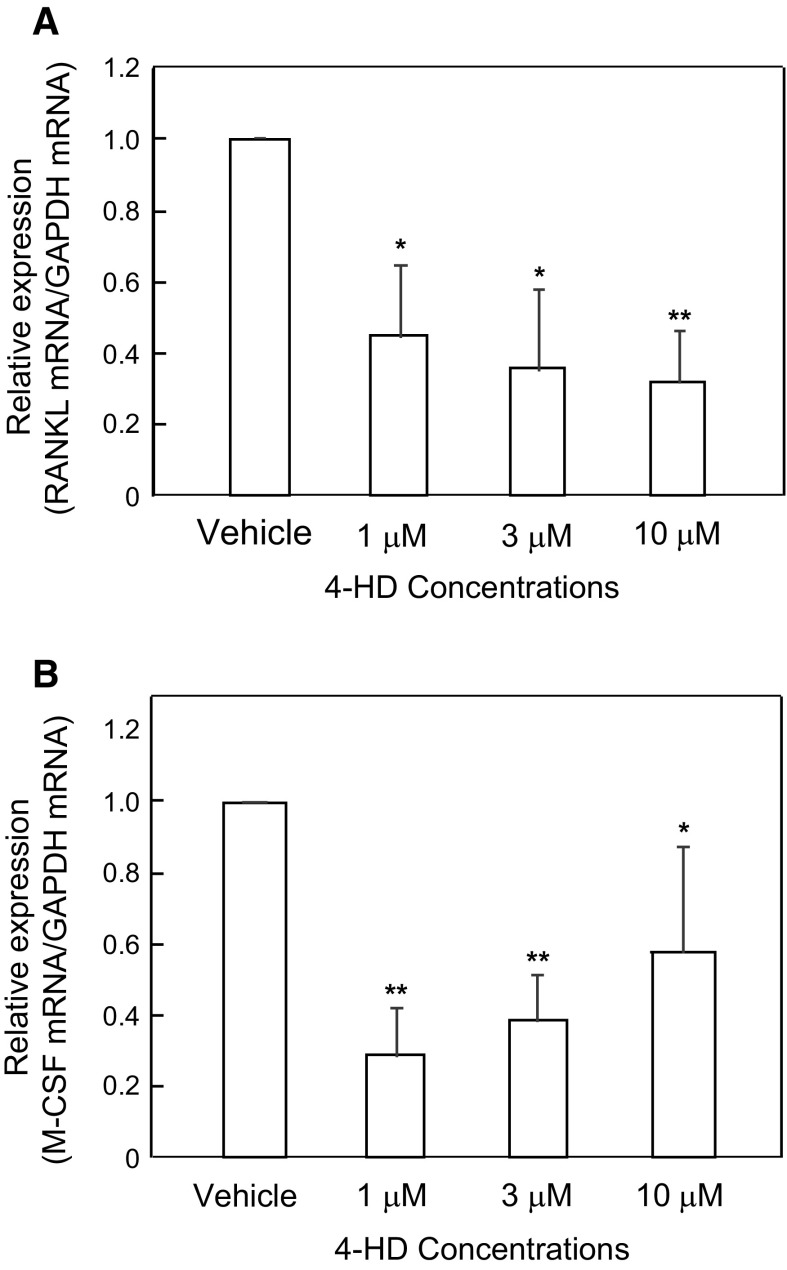
Effects of 4-HD on mRNA expression of RANKL (**a**) and M-CSF (**b**) by Real-time PCR. The RNeasy Mini Kit was used to extract RNA from ST2 cells that had been exposed to 4-HD for 2 days. The PCR conditions are described in the Materials and methods. Data are representative of results from two separate experiments. Vehicle was represented as 1

Real-time PCR revealed that 4-HD treatment of ST2 cells dose-dependently decreased RANKL mRNA expression (Fig. 4a). 4-HD also affected M-CSF mRNA expression (Fig. 4b).

### Effects of 4-HD on cultured osteoblasts

**Fig. 5:**
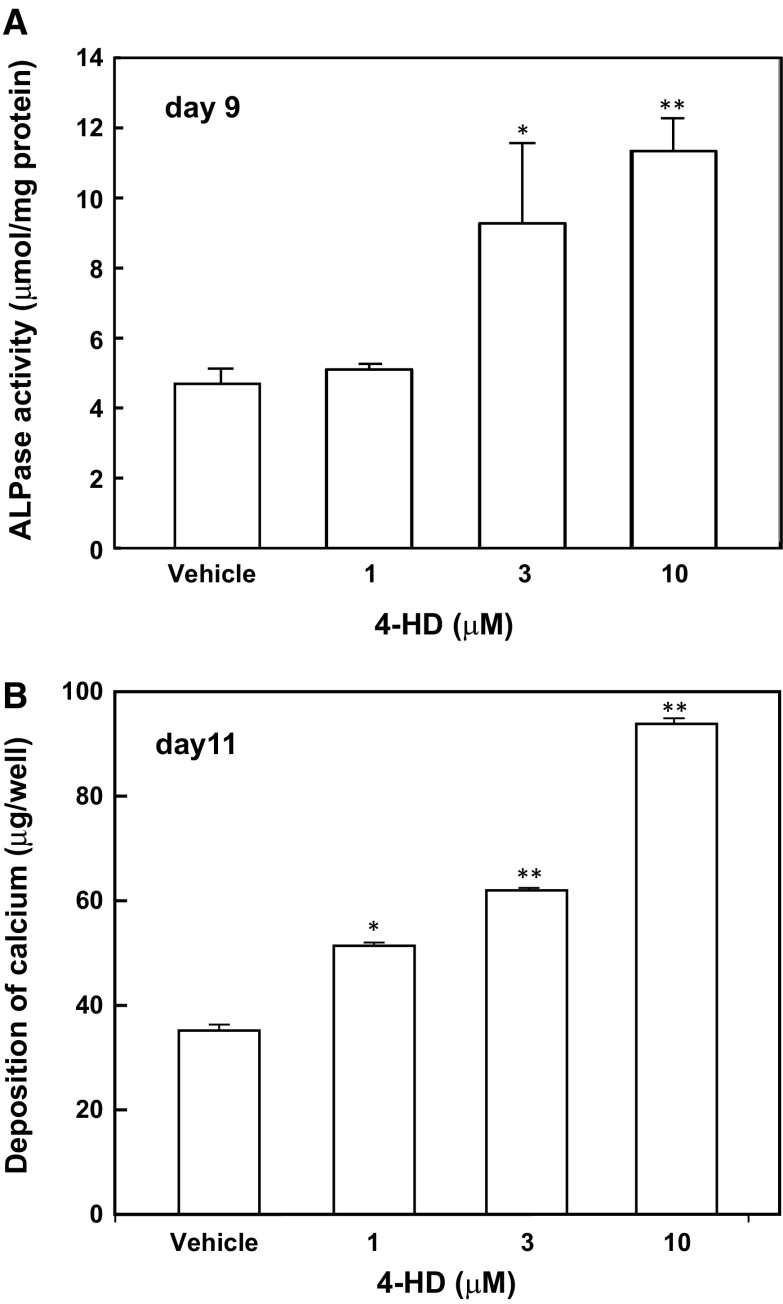
Effects of 4-HD on alkaline phosphatase activity and mineralization of osteoblasts. MC3T3-E1 cells were cultured in 12-well plates (3.8 cm^2^/well) with α-MEM containing 10% fetal bovine serum, 5 mM β-glycerophosphate, and 50 μg mL^−1^ ascorbic acid. After the cells reached confluence (day 3), 4-HD was added at various concentrations (1–10 μM) to the culture medium. Fresh medium with test compound was supplied at 3-days intervals. **a** Alkaline phosphatase activity was measured at day 9 as described in the method. **b** Deposition of Ca^2+^ ions was measured at day 11. Quantitative analysis of Ca^2+^ ions was performed as described in the method. All values represent the mean ± S.D. of the results from three wells. Data are representative of results from three separate experiments. *P < 0.05 versus vehicle (V) and **P < 0.01 versus V

To assess the effects of 4-HD on the differentiation and mineralization of MC3T3-E1 cells, we added 4-HD to the culture medium of post-proliferative cells and assayed alkaline phosphatase activity (a middle-stage marker of osteoblastic differentiation) and calcium deposition (Fig. 5). 4-HD significantly increased the activity of alkaline phosphatase in MC3T3-E1 cells on day 9 when used at 10 μM (Fig. 5a). Furthermore, as demonstrated in Fig. 5b, 4-HD dose-dependently enhanced the deposition of calcium by MC3T3-E1 cells on day 11. Exposure of MC3T3-E1 cells to 10 μM 4-HD increased the deposition of calcium by approximately 270% on day 11, relative to control cultures treated with the vehicle alone (Fig. 5b).

**Fig. 6:**
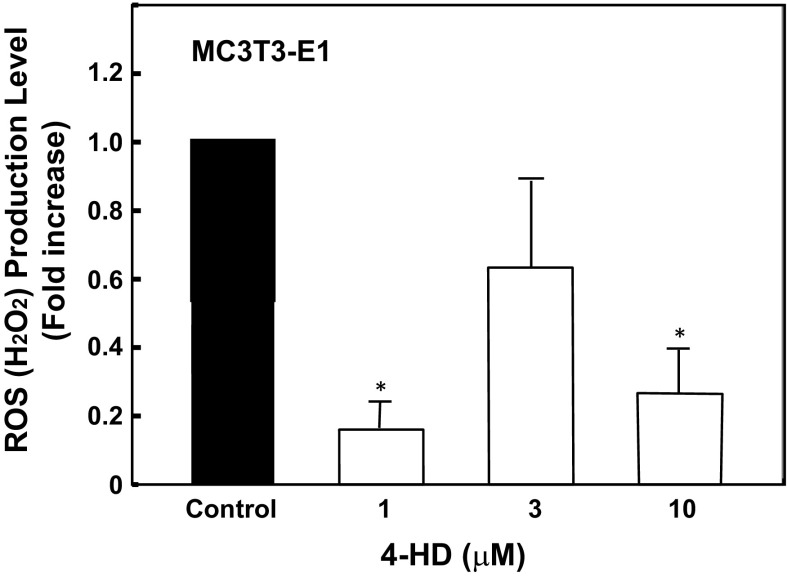
Effect of 4-HD on intracellular reactive oxygen species concentrations. Cells were pre-treated with or without 4-HD (1–10 μM) for 48 h followed by the incubation with 500 μM H_2_O_2_. Intracellular reactive oxygen species concentrations were assessed as described in the materials and methods. Data are mean ± S.E. of three to four separate experiments. **P < 0.01 versus control cells (vehicle)

It has been reported that hydrogen peroxide (H_2_O_2_) suppressed the differentiation of osteoblasts. Therefore, we examined the effects of 4-HD on H_2_O_2_ levels in MC3T3-E1 cells (Fig. 6). As shown in Fig. 6, 4-HD at 1 μM decreased H_2_O_2_ levels in cells.

## Discussion

We screened natural polyphenols for the ability to regulate the proliferation, differentiation, and function of cultured osteoclasts and osteoblasts in order to identify factors that may cause, prevent, or treat bone metabolic diseases such as osteoporosis and osteopetrosis. We had previously reported that genistein attenuates osteoclastogenesis by decreasing the levels of receptor activator NF-κB ligand mRNA in osteogenic/stromal cells (Yamagishi et al. 2001). Quercetin (Notoya et al. 2004) and curcumin (Notoya et al. 2006) have been reported to inhibit cultured osteoblast metabolism. In addition, quercetin (Woo et al. 2004) and carnosic acid (Hagiwara et al. 2015) have been found to inhibit osteoclastogenesis. Recently, we showed that the olive polyphenols oleuropein and hydroxytyrosol (Hagiwara et al. 2011) and apigenin (Goto et al. 2015) inhibit the formation of osteoclasts and attenuate bone loss in OVX mice. Thus, polyphenols regulate bone metabolism in culture via osteoclasts and osteoblasts.

In this study, we attempted to clarify the potential effects of 4-HD on bone metabolism. We found that 4-HD attenuated osteoclast formation a part through the decrease in expression of RANKL and M-CSF mRNAs and induced osteoblast differentiation markers such as alkaline phosphatase activity and calcium deposition by MC3T3-E1 osteoblasts. These findings indicate that 4-HD has properties of down-regulation of osteoclastogenesis and up-regulation of osteoblastogenesis in vitro.

It is well known that polyphenols have antioxidant properties (Rice-Evans et al. 1995). Recent reports have suggested that reactive oxygen species (ROS) play an important role in the regulation of cell proliferation, differentiation and metabolism. In particular, ROS inhibit the formation of bone by osteoblastic cells (Hosoya et al. 1998; Lee et al. 2006; Mody et al. 2001). Oxidative stress resulting in increased levels of intracellular ROS has been reported to suppress bone metabolism. Arai et al. (2007) reported that mineralization of MC3T3-E1 cells was reduced by half after a single exposure to H_2_O_2_ within the non-toxic concentration range. In addition, there have been some reports that H_2_O_2_ suppresses differentiation markers such as alkaline phosphatase activity, type I collagen gene expression, and the mineralization of osteoblastic cells (Hosoya et al. 1998; Lee et al. 2006; Mody et al. 2001). We also reported that carnosic acid attenuated H_2_O_2_ levels and osteoblastic differentiation in osteoblastic MC3T3-E1 cells (Hagiwara et al. 2015). In the present study, 4-HD significantly reduced intracellular H_2_O_2_ level of MC3T3-E1 cells. These results suggest that 4-HD induces osteoblastic differentiation a part through the decrease in intracellular H_2_O_2_ level of MC3T3-E1 cells.

Ashitaba, a perennial herb growing mainly along the Pacific coast of Japan, has been used in traditional food and medicine (Baba et al. 1998). Our results using cultured cells suggest that 4-HD may be effective on bone maintenance, if 4-HD was absorbed into the plasma. It was reported that 4-HD was quickly absorbed into the plasma after oral administration of Ashitaba extract in mice (Nakamura et al. 2012). The concentration of free 4-HD reached 1.2 ± 0.3 μM in plasma at 2 h after oral administration of Ashitaba extract at 200 mg/kg body weight. This concentration is enough to be effective on inhibition of osteoclast formation and induction of osteoblast differentiation from our results.

In conclusion, polyphenol 4-HD extracted from *Angelica keiskei* (Ashitaba) markedly inhibited the formation of multinucleated osteoclasts in culture and induced osteoblastic differentiation. These findings suggest that 4-HD may provide insights into the development of tools useful for the prevention and treatment of osteoporosis.
